# Datopotamab deruxtecan: a new era in targeted therapy for metastatic breast cancer

**DOI:** 10.1097/MS9.0000000000003857

**Published:** 2025-09-12

**Authors:** Maryam Mobashir, Huda Tarique, Shakeel Ahmad, Iffa Habib, Areeba Inam, Ihsan Qamar, Waseem Sajjad

**Affiliations:** a Department of Medicine, Dow University of Health and Sciences, Karachi, Pakistan; b Department of Medicine, King Edward Medical University, Mayo Hospital, Lahore, Pakistan; c Department of Medicine, Saidu Medical College, Swat, Pakistan; d Department of Medicine, Jinnah Sindh Medical University, Karachi, Pakistan; e Department of Medicine, Ayub Medical College, Abbottabad, Pakistan; f Department of Medicine, Spinghar University, Kabul, Afghanistan

**Keywords:** breast cancer, datopotamab deruxtecan, metastatic cancer, oncology, targeted therapy

## Abstract

Datopotamab deruxtecan (Datroway) is approved by the FDA to treat hormone receptor-positive, human epidermal growth factor receptor 2-negative breast cancer that is metastatic or incurable. This antibody-drug combination consists of a linker, the cytotoxic compound DXd, and a monoclonal antibody (mAb) that targets TROP2. Following internalization facilitated by the mAb’s binding to TROP2 on cancer cells, DXd is released, causing cell death. Dato-DXd outperformed traditional chemotherapy in the TROPION-Breast01 study. With better efficacy than conventional treatments, it offers a possible alternative for individuals who have not responded to previous endocrine or chemotherapy therapies. This manuscript reviews the mechanisms of action, clinical efficacy, safety profiles, and trials of Dato-DXd and T-DXd, highlighting their transformative potential and positioning in current breast cancer treatment algorithms.

On 17 January 2025, the US FDA approved the Datopotamab deruxtecan-dlnk (Datroway) for advanced metastatic hormone receptor (HR)-positive, human epidermal growth factor receptor 2 (HER2)-negative breast cancer, marking a significant milestone in oncologic therapeutics^[1]^. With greater efficacy than traditional treatments, the medication presents a potential substitute for individuals who have not responded to prior treatment options.

Cancer management initially involved surgery, chemotherapy, and radiotherapy largely dependent upon the survival and quality of life of the patients. Recent advancements in immunotherapy as a treatment modality for various tumors have been gaining prominence. A recent study analyzing the trends of immunotherapy demonstrated a significant shift toward neoadjuvant settings, showing a substantial rise in related research compared to adjuvant therapy^[2]^. However, a considerable subset of patients with metastatic HR+/HER2− breast cancer continue to experience progression after standard therapies. This highlights the need for innovative targeted approaches such as antibody–drug conjugates (ADCs). In this context, Datroway has emerged as a promising therapeutic option.

An ADC called Datroway is intended to destroy cancer cells only while leaving healthy cells unharmed. It combines Deruxtecan, a potent cytotoxic medication, with Datopotamab, a monoclonal antibody that targets TROP2, a single-pass transmembrane protein that is highly expressed in various epithelial cancers, and its overexpression is highly linked to poor prognosis. When the drug binds to TROP2, it delivers its payload directly into cancer cells, leading to apoptosis, DNA damage, and tumor growth inhibition with minimal damage to healthy tissues^[3]^.

TROPION-Breast01, a multicenter, open-label, randomized trial, was conducted to assess the drug’s efficacy compared to chemotherapy. The trial included 700 patients who were ineligible for endocrine therapy, had undergone one or two lines of chemotherapy, and could not have their tumors surgically removed. Evaluated by blinded independent central review, following RECIST v1.1, and overall survival (OS), the primary efficacy outcome measures included progression-free survival (PFS). Moreover, the OS data was not yet fully mature at the time of the primary analysis^[4]^. Other efficacy outcomes comprised objective response rate and duration of response^[1]^. The exploratory endpoints pave the way for future research on the association of TROP2, tumor-derived biomarkers, and ctDNA with clinical outcomes and tolerability to datroway^[4]^.

Sacituzumab govitecan (SG) is another TROP2-targeting ADC approved by the FDA. In comparison with datroway, both drugs have a similar risk of cessation without advancement and toxic mortality. However, adverse effects observed with SG included neuropathy, diarrhea, alopecia, fatigue, and hematological toxicities such as anemia and neutropenia. Stomatitis, interstitial lung disease, ocular, and infusion reactions were specific to datroway. Overall, no significant difference in efficacy was observed^[5]^.

According to available data, ADCs are safe and effective for breast cancer patients, demonstrating better OS and PFS combined with a manageable safety profile. Since aging causes physiological changes, the pharmacokinetics and pharmacodynamics of ADCs in older individuals need to be carefully assessed. With HER2- or TROP2-targeted ADCs, changes in hepatic clearance, protein binding, and renal elimination can impact ADC metabolism and increase vulnerability to toxicities. The recommended dose of datroway is 6 mg/kg given as an intravenous infusion once every 3 weeks until disease progression or intolerable toxicity^[4]^.

In conclusion, additional research needs to be conducted to explore the drug’s impact on various biomarkers. Moreover, several drugs’ comparisons should be made simultaneously to investigate their effectiveness and side effects, and prophylaxis should be recommended to mitigate adverse effects.

This work has been reported according to TITAN guidelines^[6]^.

## Data Availability

Not applicable.
